# Physicochemical and Photocatalytic Properties of 3D-Printed TiO_2_/Chitin/Cellulose Composite with Ordered Porous Structures

**DOI:** 10.3390/polym14245435

**Published:** 2022-12-12

**Authors:** Lei Li, Jingdan Li, Hao Luo, Shengjuan Li, Junhe Yang

**Affiliations:** School of Materials and Chemistry, University of Shanghai for Science and Technology, 516 Jungong Road, Shanghai 200093, China

**Keywords:** 3D printing, cellulose, chitin, NMMO, TiO_2_, 3D porous photocatalyst, interconnected porous structures, MB degradation

## Abstract

In this study, we printed three-dimensional (3D) titanium dioxide (TiO_2_)/chitin/cellulose composite photocatalysts with ordered interconnected porous structures. Chitin microparticles were mixed with cellulose in the N-methylmorpholine-N-oxide (NMMO) solution to prepare the printing “ink”. TiO_2_ nanoparticles were embedded on the chitin/cellulose composite in the NMMO removal process by water before the freeze-drying process to build the 3D cellulosic photocatalysts with well-defined porous structures. The 3D-printed TiO_2_/chitin/cellulose composites were characterized by X-ray Diffraction (XRD), Fourier Transform Infrared Spectroscopy (FTIR), Scanning Electron Microscopy (SEM), and Energy Disperse Spectroscopy (EDS). The XRD and FTIR analyses showed that chitin had an interference effect on the crystal regeneration of cellulose and resulted in a large amount of amorphous phase. The SEM images show that the printed cellulosic strands had a hollow structure, and the EDS analysis showed that TiO_2_ nanoparticles were embedded on the chitin/cellulose composite surfaces. In the photocatalytic degradation process of methylene blue (MB) dye in an aqueous solution, the TiO_2_/chitin/cellulose 3D composite photocatalysts demonstrated efficient MB degradation activities with excellent reusability and stability, in which the chitin content performed the function of adjusting the MB degradation efficiency.

## 1. Introduction

There is growing interest in composite materials that are fabricated by combining inorganic materials and organic polymers. Such inorganic/polymer composite materials perform tailored functions that are improved by the characteristics of each component, which could open up possibilities for applications in various industries, such as adsorption [1], catalysts [2], and sensors [3]. As a result of their environmental effects, abundance, and material properties, natural polymers, such as cellulose and chitin, are extremely useful [4]. Cellulose and chitin are the two most abundant polymers and have become advanced functional materials with great potential in multiple areas. Cellulose and chitin have similar chemical structures with various functional groups. Cellulose has a molecular structure comprising linear anhydro-D-glucose unit (AGU) rings, which are joined via β (1 → 4) linkages, while chitin is composed of N-acetyl-D-glucosamine residues. Cellulose and chitin have abundant hydroxyl groups and active sites to facilitate the adsorption of dyes dissolved in water [5]. As such, cellulose and chitin appear to have good miscibility and can be well-incorporated to improve mechanical properties and produce multiple advanced functions as compared to homopolymers [6].

Recently, cellulosic inorganic/polymer composite materials of great interest have been developed [7,8,9,10,11,12]. To invent new functional materials by exploiting the excellent properties of cellulose, first, the dissolving problem must be addressed. Harsh and inefficient traditional viscose routes are not suitable for constructing advanced composite structures. Fortunately, several dissolving systems with moderately polluting and nonpolluting processes have been reported in the last several decades, which provide better ways to modify cellulose through the use of inorganic materials [13,14,15]. Composite materials made of inorganic/cellulose, currently in the form of particles [16], films [9], and aerogels [17,18], have promising applications in various fields, such as adsorbent, catalyst supports, textiles, electronic devices, and tissue engineering. Among cellulose products, cellulose aerogels have cross-linked three-dimensional (3D) structures with low densities, high porosities, large specific surface areas, and abundant hydroxyl groups on their interfaces. Therefore, these matrices are useful in the fabrication of inorganic/polymer composite materials [18,19,20,21]. In this way, cellulose composite aerogels can produce improved multifunctional characteristics, including mechanical, optical, and catalytic properties for various promising applications. In addition, such 3D aerogels can overcome the difficulty of inorganic powder recycling, can be made into portable devices, and do not exhibit spontaneous agglomeration of inorganic particles on a two-dimensional surface of film materials [18]. As such, they can be utilized in multitudinous fields, e.g., large-scale pollutant treatments.

TiO_2_ has been one of the most prominent inorganic materials since its photocatalyst ability was discovered. As the first photocatalyst material for practical applications, TiO_2_ has been utilized to fabricate pollutant treatment photocatalysts [22,23,24], self-clean materials [25,26], and antibacterials [27,28,29] that are low-cost and exhibit nontoxicity, high photocatalytic efficiency, and long-term stability. As new novel materials, TiO_2_/cellulose composite beads and films have become a research focus [9,16,30,31,32,33,34]. Although one-/two-dimension (1/2D) nanoparticles/nanosheets can provide an adsorber with excellent adsorption performance due to the abundance of exposed adsorption sites, it is difficult to separate nanoadsorbents after pollutant removal from wastewater. The nanoparticles/nanosheets are toxic and can cause secondary pollution and kill cells that they penetrate [35]. Furthermore, the reusability and practical applications of such 1/2D adsorbents are limited. Regarding such problems, a 3D structure is one feasible strategy to develop wastewater treatments [36]. TiO_2_/cellulose 3D composite materials are attracting a great deal of attention from researchers, as they retain the excellent properties of TiO_2_ and cellulose and provide high porosity, excellent mechanical properties, high separability, and reusability [37,38]. For example, Yue Jiao et al. simply immersed cellulose hydrogels in an anatase TiO_2_ nanosol and subsequently removed water to produce TiO_2_/cellulose aerogels [18]. They found that such composite aerogels had high photocatalytic efficiency for dye degradation and maintained their shapes well during the dye degradation process. Currently, the methods for fabricating inorganic/cellulose 3D structures include direct freeze drying or supercritical CO_2_ drying of bulk hydrogels; however, the corresponding structures do not normally have controlled interconnected macropores, which enhance the adsorption of materials.

Chitin is the second most abundant natural polymer on earth and can be treated as a cellulose derivative polysaccharide. Chitin is nitrogenous, rigid, inelastic, and has inferior thermal and mechanical properties. Its high porosity, low density, renewability, and biodegradability means it is utilized in chelating agents, wound-healing agents, and adsorbers for dye removal from wastewater, which have been extensively investigated [5]. Nevertheless, chitin’s inferior mechanical properties restrict its widespread application [39]. On one hand, the synthetic chitin/cellulose composite may greatly improve the stability of chitin products. On the other, the increased content in the chitin/cellulose composite can decrease the crystallinity of cellulose, which increases the ability of molecules to access the inner regions of cellulose and enhance adsorption [40]. It is also desirable to produce macro/micropores on chitin samples to improve adsorption efficiency.

The 3D printing technique, a novel, fast-growing manufacturing method that exhibits efficient and multifunctional performance, can be used to construct presetting interconnected porous structures of inorganic/cellulose composite materials. However, to date, no 3D printing inorganic/cellulose composite structures or 3D printing with pure chitin have been reported. With the aid of a computer system, predesigned complex 3D hierarchical structures can be fabricated in a layered printing process [41]. The structure size can vary to satisfy customers’ individual requirements, with feature sizes in both the microscale (0.1–100 μm) and mesoscale (>100 μm). The rheological behavior of the printing materials and the printing parameters, e.g., the printing velocity, temperature, and pressure extrusion, are main factors that affect the printing process [42].

In this paper, we report a 3D-printed TiO_2_/chitin/cellulose composite photocatalyst with an ordered interconnected porous structure. Cellulose and chitin are good coadsorbers of organic dyes in water. Fine chitin microparticles were added to adjust the composite photocatalyst’s properties because chitin can decrease the crystallinity of polymer blends and improve the composite’s thermal stability [6], which occurs as a result of hydrogen bonding between cellulose and chitin. The lower crystallinity of cellulose may improve the adsorption ability of the TiO_2_/cellulose composite photocatalyst in an aqueous environment [43]. The photocatalytic degradation process of methylene blue (MB), a synthetic cationic dye, in aqueous solution was used to demonstrate the potential of such 3D-printed TiO_2_/chitin/cellulose composite materials for organic pollutant removal.

## 2. Materials and Methods

### 2.1. Materials

Dissolving pulps for cellulosic 3D printing were kindly supplied by Shandong Yamei Technology Co. Ltd. (Binzhou, China). The dynamic viscosity of the dissolving pulps is 18–20 mPa·s. Raw chitin flakes, N-methylmorpholine-N-oxide (50 wt% H_2_O) (NMMO), TiCl_3_ solution (15 wt% TiCl_3_ in 10 wt%HCl aqueous solution), and urea were supplied by Beijing HWRK Chem Co. Ltd. (Shanghai, China). All reagents were of analytic reagent grade.

### 2.2. Preparation of 3D-Printed Photocatalysts

#### 2.2.1. Preparation of TiO_2_ Nanoparticles

The TiO_2_ nanoparticles were prepared by mixing the aqueous TiCl_3_ solution with urea ((NH2)_2_CO) followed by a hydrothermal treatment at 190 °C [44]. A total of 7 g of urea and 21.5 mL of 15 wt.% TiCl_3_ solution were added to 25 mL distilled water, and then the mixed solution was poured into a Teflon container. After nitrogen flushing for 10 min, the Teflon container was sealed in a SUS 314 stainless steel autoclave. The autoclave was first heated at 90 °C for 1 h, and then heated at 190 °C for 2 h. According to this recipe, the final pH value of the solution was 7. The synthetic powder in the Teflon container was collected by centrifugation, washed with distilled water and acetone, respectively, and then vacuum dried at 80 °C overnight.

#### 2.2.2. Dissolution of Cellulose in NMMO

Chitin flakes were treated with weak acids to remove inorganic components and subsequently NaOH to remove proteins. Thereafter, they were pulverized through a mill blade grinder with rotation velocity higher than 30,000 rpm before being sifted through a 600-mesh sieve. In this way, chitin particles with diameters less than 24 μm were collected. Subsequently, 0.5, 1, or 2 g of the chitin particles were dispersed into 100 mL NMMO (50 wt% H_2_O) at 90 °C. The temperature was maintained via immersion of the beaker in a silicone oil bath and monitored with a thermometer. Subsequently, the chitin/NMMO suspension solution was continuously stirred to maintain its homogeneity when the chitin particles swelled in NMMO. After stirring for approximately 2 h at 90 °C, the temperature of the solution was increased to 115 °C, at which point a mass of 5 g of dissolving pulps was added. Another hour of heating turned the chitin/cellulose/NMMO suspension solution into a yellow viscous solution without an observed trace of cellulose fibers. Thereafter, the temperature of the chitin/cellulose/NMMO solution was reduced to 70 °C for subsequent 3D printing.

#### 2.2.3. 3D Printing of TiO_2_/Chitin/Cellulose

The printing was conducted using the 4th 3D Bioplotter^TM^ (EnvisionTEC, GmbH, Gladbeck, Germany) with a high-temperature printing function. The chitin/cellulose/NMMO solution was loaded into a stainless printer cartridge and fixed on a heating system, which was designed to bear 250 °C. Cube models, each face being 10 mm square, were designed using the Bioplotter CAD/CAM software and were plotted layer-by-layer at 70 °C through one 400 μm (22 G) diameter extrusion nozzle. The gas extrusion pressure was 2 bar and the speed of the dispensing unit was 20.0 mm/s. The extruded chitin/cellulose/NMMO gel quickly solidified after it was extruded from the cartridge; therefore, it maintained the printed structure well. Water was added dropwise to regenerate cellulose during the printing process, and to stabilize the 3D interconnected porous structures. In order to completely remove the NMMO solvent and embed TiO_2_ nanoparticles on the chitin/cellulose photocatalyst, the printed composites were immersed into 150 mL milli-Q water with 0.3 g TiO_2_ nanoparticles at 90 ℃ for 6 h, which resulted in TiO_2_/chitin/cellulose composite hydrogels with ordered interconnected pores. The composite hydrogels were fed into an ultra-low temperature freezer at −70 °C, and then they were freeze dried to form 3D-printed, ordered, interconnected porous photocatalysts.

### 2.3. Characterization and Instruments

Microstructures and surface chemical compositions were characterized by a Scanning Electron Microscope (SEM, Quanta^TM^ 450 FEG, FEI, Graz, Austria) equipped with an Energy-Dispersive X-ray Spectrometer (EDS). Crystal structures were characterized via X-ray Diffraction (XRD, Bruker D8 Advance) operating with Cu K*α* radiation (*λ* = 1.5418 Å) at a scan rate (2*θ*) of 4°/min ranging from 5° to 60°. Nitrogen adsorption–desorption measurements were taken on a Micromeritics Tristar 3020 at −196 °C under continuous adsorption conditions. Brunauer–Emmett–Teller (BET) and Barrett–Joyner–Halenda (BJH) analyses were performed to measure the specific surface area, the pore volume, and the pore size distribution of mesopores in the printed photocatalysts.

### 2.4. Photocatalytic Degradation of Methylene Blue (MB)

To investigate the photocatalytic ability of the 3D-printed TiO_2_/chitin/cellulose composite photocatalysts, different ratios of cellulose to chitin in the samples were employed in the photocatalytic degradation tests of the cationic MB dye under UV light irradiation. The concentration of MB solution could be determined on an ultraviolet-visible spectrophotometer by detecting the absorbance intensity of the UV/visible absorbance peak of MB located at 664 nm. The MB concentrations were measured at given irradiation time intervals. The changes demonstrated the photocatalytic activity of the 3D-printed TiO_2_/chitin/cellulose composite photocatalysts.

Three groups of photocatalysts with various chitin contents were utilized: 0.5, 1, and 2 g of chitin particles in each of the three groups with 0.3 g of TiO_2_ nanoparticles and 5 g of cellulose, which are termed TiO_2_/chitin/cellulose (10/1), TiO_2_/chitin/cellulose (5/1), and TiO_2_/chitin/cellulose (5/2), respectively. Moreover, the pure printed cellulose and TiO_2_/cellulose composites were also used to elucidate the degree of photocatalytic activity of the 3D-printed composite photocatalysts.

After preparing MB dye solution with a concentration of 50 mg/L, the printed composite photocatalyst (10 mm square cubes) was added to 200 mL of MB dye solution for each group. Before the irradiation process, all printed samples were immersed in the MB solution and stirred for 0.5 h in a totally dark environment to reach a supposed adsorption equilibrium. Thereafter, the UV source (mercury lamp, 300 W, and 365 nm) was turned on at a distance of 10 cm from the MB solution. The concentration of MB solution was measured intermittently in order to monitor the photocatalytic degradation process.

## 3. Results

### 3.1. Characterization of 3D-Printed TiO_2_/Chitin/Cellulose Photocatalysts

#### 3.1.1. Morphological Studies of the Printed TiO_2_/Chitin/Cellulose Photocatalysts

**Figure 1:**
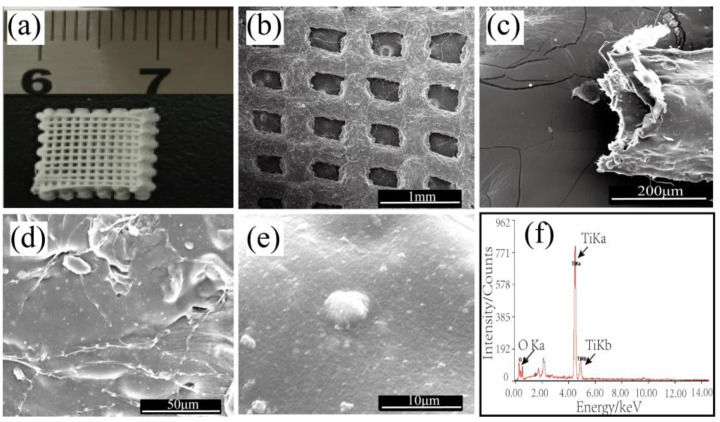
Photograph (**a**) and SEM images of the TiO_2_/chitin/cellulose photocatalyst with a magnification of 100× (**b**), 2000× (**d**), and 10,000× (**e**). (**c**) shows the hollow structure of the printed strands. (**f**) is one EDS spectrum for the strand surface, which indicates the presence of an abundance of titanium compounds.

The optical photograph and SEM images in Figure 1a,b demonstrate that the 3D-printed TiO_2_/chitin/cellulose photocatalysts exhibited a 90°orientation difference between two successive layers and maintained the 3D ordered interconnected porous structures after the freeze-drying process. The micropore size of the printed structure was 500 μm, as shown in Figure 1b. Figure 1c shows that the strand extruded from a nozzle had a hollow structure. The hollow structure may be produced during the removal of NMMO solvent and the freeze-drying process. NMMO was replaced by water in the printed cellulose/chitin sample, which happened in the traditional NMMO removal process to regenerate cellulose. The chitin/cellulose samples maintained their shapes in the freeze-drying process, with many hollow strands generated when the water was removed from the samples. Figure 1d,e indicates that the surface of the strands was rough, with large amounts of evenly distributed nanoparticles embedded in them. In addition, apart from the common C and O elements, the EDS spectrum of the 3D-printed photocatalyst exhibited a strong peak assigned to Ti (Figure 1f), which indicates the presence of plentiful titanium compounds.

#### 3.1.2. XRD Analyses

**Figure 2:**
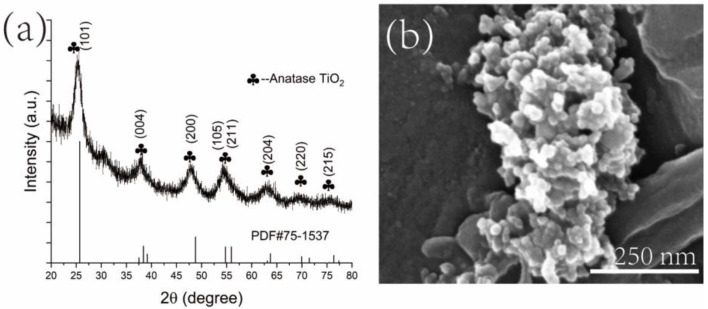
(**a**) XRD profile of the synthetic TiO_2_ nanoparticles produced using a hydrothermal method at 190 °C for 2 h using urea and TiCl_3_; (**b**) SEM image of the synthetic TiO_2_ nanoparticles.

X-ray diffraction analysis was performed to determine the crystal phase of the 3D-printed composites with various cellulose/chitin ratios. First, the crystal phase of synthetic TiO_2_ was determined using XRD. Figure 2a shows the XRD pattern of the synthetic TiO_2_ nanoparticles. The peaks, indexed as 2*θ* = 25.7°, 3.8.4°, 48.8°, 55.9°, 63.7°, 71.5°, and 76.4°, were assigned to an anatase TiO_2_ phase (PDF#75-1537), corresponding to the (101), (004), (200), (211), (204), (220), and (215) crystal plane. The crystallite size of the anatase TiO_2_ was calculated using the Scherrer Equation: D = K*γ*/(B*cosθ), where K is the Scherrer constant (0.89) when B is taken as peak width at half height; *γ* is the wavelength of X-ray (1.540456 Angstrom). The crystalline size of the synthesized TiO_2_ nanoparticles was approximately 5.1 nm according to the calculation. The SEM image of the TiO_2_ nanoparticles is presented in Figure 2b. It shows that the average size of the nanoparticle was approximately 15.4 ± 4.2 nm, which is slightly larger than the calculated crystallite size from the Scherrer Equation.

**Figure 3:**
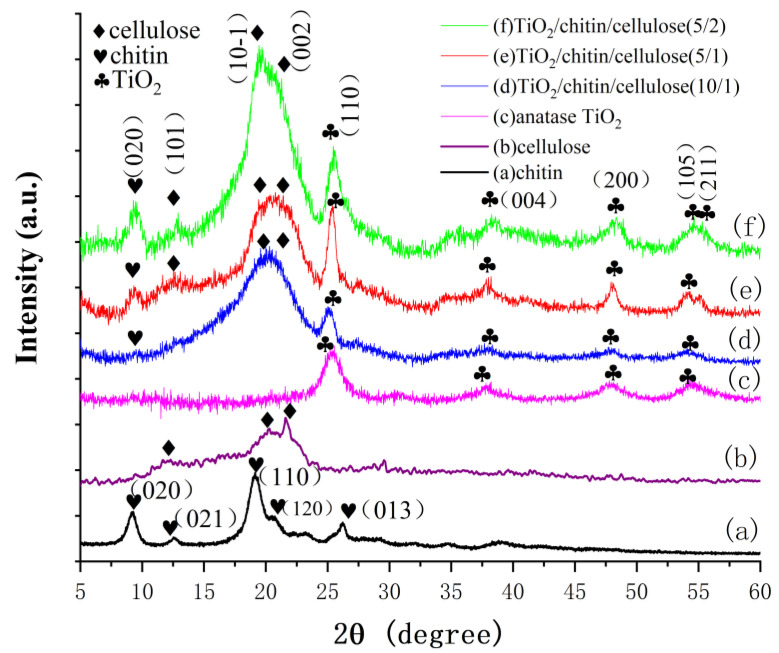
XRD profiles for printed composites, including (a) chitin, (b) pure cellulose, and (c) TiO_2_ nanoparticles. Composites with 0.3 g TiO_2_ and a weight ratio of cellulose to chitin of (d) 5 g: 0.5 g, (e) 5 g: 1 g, and (f) 5 g: 2 g.

The XRD patterns of the chitin, cellulose, TiO_2_, and TiO_2_/chitin/cellulose composites with cellulose/chitin ratios of 5/2, 5/1, and 10/1 are shown in Figure 3. The XRD pattern (a) of pulverized chitin particles has typical *α*-chitin crystal peaks at 2*θ* = 9.39°, 12.67°, 19.72°, 20.73°, 23.33°, and 25.90°, corresponding to the (020), (021), (110), (120), (130), and (013) crystal indexes, respectively [45]. The curve (b) for the 3D-printed pure cellulose matrix has typical XRD profile peaks at 2*θ* = 12.3° (101), 20.3° (10-1), and 21.6° (002), indicating a cellulose II phase [46,47]. The crystallinity index of the 3D-printed pure cellulose was assessed through the intensity of the (002) peak and the intensity of the pattern at 18.14° (implying an amorphous phase through deconvolution) [48,49]. The pure cellulose sample had a crystallinity of 61.3%. In addition, the XRD pattern (c) of TiO_2_ in Figure 3 shows the diffraction peaks of an anatase TiO_2_ phase (2*θ* = 25.3°, 37.8°,48.8°, and 55.9°). The curves (d), (e), and (f) in Figure 3 show that the peaks at 25.3° could be clearly distinguished as the characteristic peak of anatase TiO_2_, demonstrating the successful incorporation of TiO_2_ nanoparticles on the chitin/cellulose matrices. With the enhancement of the chitin content in the composite, the characteristic peak of chitin at 9.39° was observed in the XRD pattern, as shown in (d), (e), and (f). The peak at 19.72°, belonging to chitin, may be involved with the peak of cellulose at 20.3°. The crystallinities of cellulose in the composites were calculated. They decreased with increasing chitin content, from approximately 61.3% for pure cellulose to 57.1% for TiO_2_/chitin/cellulose (10/1), 56.6% for TiO_2_/chitin/cellulose (5/1), and 55.7% for TiO_2_/chitin/cellulose (5/2). This implies that the addition of chitin during cellulose dissolution in NMMO increased the nanocrystalline cellulose regions [40,50].

#### 3.1.3. BET Analyses

**Figure 4:**
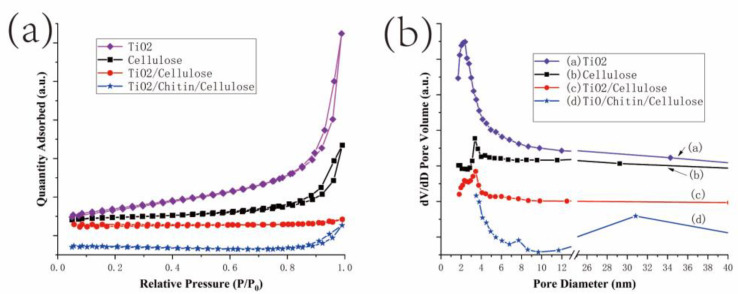
(**a**) N2 adsorption–desorption isotherms for TiO_2,_ 3D-printed cellulose, TiO_2_/cellulose, and TiO_2_/chitin/cellulose. (**b**) The corresponding pore size distribution of TiO_2_ nanoparticles, 3D-printed cellulose, TiO_2_/cellulose, and TiO_2_/chitin/cellulose.

Nitrogen adsorption–desorption measurements were used to estimate the specific surface area and porosity characteristics of the 3D-printed TiO_2_/chitin/cellulose composites and the synthetic TiO_2_ nanoparticles. The data of the surface area, pore volume, and pore size are listed in Table 1. The adsorption–desorption isotherms of TiO_2_, the 3D-printed pure cellulose, TiO_2_/cellulose, and TiO_2_/chitin/cellulose materials are shown in Figure 4a and the pore size distributions are given in Figure 4b. The specific surface area of the synthesized TiO_2_ was 50 m^2^/g. The specific surface areas of the printed pure cellulose, TiO_2_/cellulose, and TiO_2_/chitin/cellulose in the present study were 15, 1, and 3 m^2^/g, respectively, according to the BET analysis, which were significantly lower than those in the reported data (50~420 m^2^/g) for regenerated cellulose aerogel from NMMO via supercritical CO_2_ drying [51,52,53]. Although TiO_2_/cellulose had a low specific surface area in the present work, the addition of chitin significantly increased its specific surface area. The pore size distribution profiles of TiO_2_, the printed pure cellulose, TiO_2_/cellulose, and TiO_2_/chitin/cellulose exhibit obvious peaks for their pore sizes at 2.4, 3.4, 2.3/4.8, and 7.7/30.8 nm in Figure 4b, respectively. It is worth noting that no pore size peak was observed for TiO_2_/chitin/cellulose at a range smaller than 5 nm in Figure 4b.

#### 3.1.4. FT-IR Spectra of 3D-Printed TiO_2_/Chitin/Cellulose Photocatalysts

**Figure 5:**
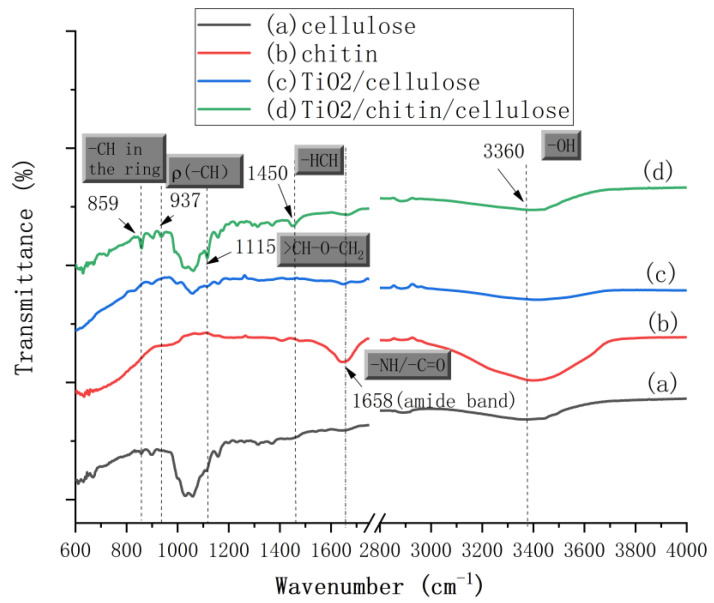
FT-IR spectra curves of the 3D-printed photocatalysts composed of (a) cellulose (5 g) (black), (b) chitin (red), (c) TiO_2_ (0.3 g)/cellulose (5 g) (blue), and (d) chitin (2 g)/cellulose (5 g) (green).

The FT-IR spectra for the 3D-printed cellulosic photocatalysts and the pure chitin particles are shown in Figure 5. The FT-IR spectrum of the printed pure cellulose (Figure 5 curve (a)) shows characteristic peaks of the hydrogen bond (-OH) in the range of 3000–3700 cm^−1^ and the stretching vibration of the methylene group (-CH_2_) in the range of 2800–3000 cm^−1^, which also appeared in the FT-IR spectra of chitin, TiO_2_/cellulose, and TiO_2_/cellulose/chitin samples. The FTIR spectrum of chitin (Figure 5 curve (b)) has an intense and broad peak centering at 1658 cm^−1^, comprising the -C=O stretching and -NH bending vibration signals in the amide band of a typical *α*-chitin structure [54]. However, four intensive peaks at 859, 937, 1115, and 1450 cm^−1^ were found after the chitin was added to the TiO_2_/cellulose composite (curve (d)). These peaks were not observed in the spectrum of the TiO_2_/cellulose composite. Therefore, they were not caused by the addition of TiO_2_ nanoparticles, but from the interaction between cellulose and chitin. The peak at 859 cm^−1^ was assigned to the C-H stretching of the disturbed rings of cellulose/chitin, while the peaks at 937 and 1115 cm^−1^ were assigned to the deformation of the -C-H- bending and >CH-O-CH_2_ stretching vibration outside of the cellulose/chitin ring, respectively [55,56]. The peaks at 1450 cm^−1^ were attributed to the -HCH- scissoring bending vibration of the cellulose amorphous region caused by crystal modification by chitin, which was not shown or was extremely weak in the spectra of the pure cellulose and chitin samples. This clearly indicated that the addition of chitin particles into the cellulose/NMMO solution weakened the intramolecular hydrogen bonding (formed by O and C6) and changed the regeneration process of cellulose [57].

### 3.2. Mechanical Tests

**Figure 6:**
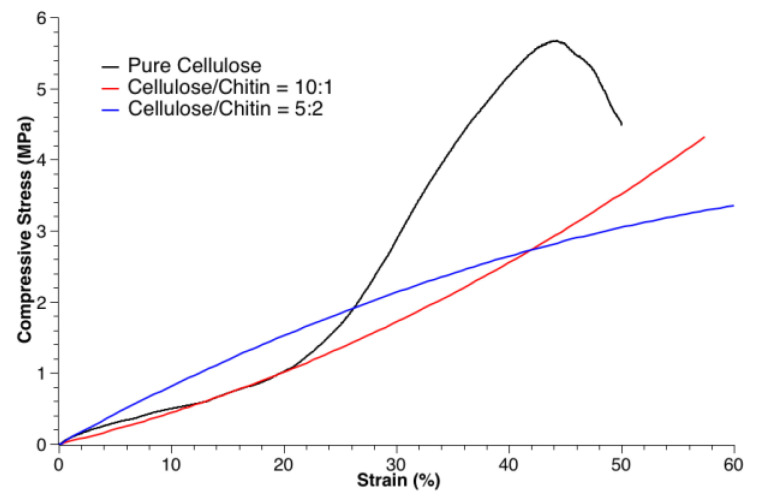
Compressive stress–strain curves for the 3D-printed products of pure cellulose (black), cellulose/chitin = 10:1 (red), and cellulose/chitin = 5:2 (blue).

The mechanical characteristics of the 3D-printed chitin/cellulose products in a cubic shape (10 mm square) after freeze drying were studied. The compressive stress–strain curves for the TiO_2_/chitin/cellulose (10/1), TiO_2_/chitin/cellulose (5/2), and pure cellulose products are presented in Figure 6. It was observed that the composite products have similar mechanical characteristics. However, the addition of chitin slightly lowered the toughness of the pure cellulose product, although it exhibited better strain before collapsing. It is worth noting that cellulose products 3D-printed from an NMMO solution can withstand compressive stress well [58].

### 3.3. Photocatalytic Tests

**Figure 7:**
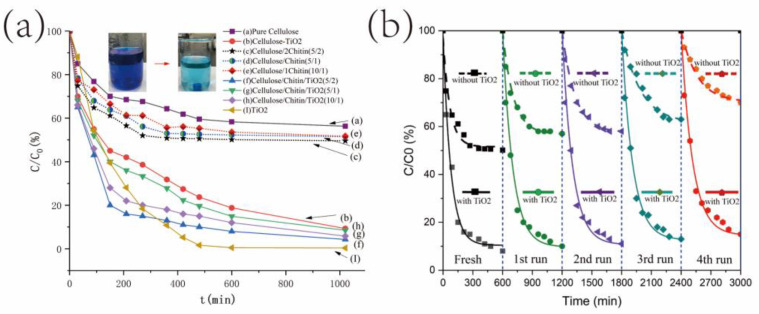
(**a**) Photodegradation curves of MB solution for 3D-printed cellulosic composites and photocatalysts under UV irradiation of pure cellulose, pure TiO_2_, TiO_2_/cellulose, TiO_2_/chitin/cellulose with 0.5 g, 1 g, 2 g of chitin added to 5 g cellulose, and chitin/cellulose with 0.5 g, 1 g, 2 g of chitin added to 5 g cellulose, respectively; (**b**) 5 cycles of photodegradation of MB using the TiO_2_/chitin/cellulose (2 g chitin to 5 g cellulose) sample and the chitin/cellulose (2 g chitin to 5 g cellulose, without TiO_2_) sample.

All the as-prepared TiO_2_/chitin/cellulose photocatalysts for the photodegradation tests were 10 mm square cubes. Both cellulose and chitin are good co-adsorbers. In order to reduce the interference of adsorption in the photocatalytic activity measurements, all printed samples were immersed in MB solution and stirred for half an hour in a dark environment to reach adsorption equilibrium before the irradiation process. Only the adsorption of MB from aqueous solution into the printed composites occurred in the first half an hour. The photographs inset in Figure 7a demonstrate the efficiency of MB removal from water with the TiO_2_/chitin/cellulose composite sample. It was observed that the color of all printed samples changed from white to nattier blue, while the color of the MB solution stayed the same in this period. This indicated that the adsorption activity was not powerful enough to remove MB from the aqueous solution in a short time. Subsequently, in the UV irradiation process, the dark blue color of the MB solution faded progressively. This phenomenon suggested that the printed TiO_2_/chitin/cellulose photocatalysts have promise for use in certain promising photocatalytic applications, such as wastewater treatment to remove organic dye. Moreover, all samples maintained their defined interconnected porous structures well in MB solution without collapsing during the UV irradiation process, which demonstrates the possibility of recycling the photocatalysts.

The concentration decreasing curves of MB are shown in Figure 7a. The curve in (a) shows the lowest MB-removal capability of the 3D-printed pure cellulose sample, which simply occurred via adsorption, while the curve in (b) indicates a significant enhancement of the MB-removal rate due to TiO_2_ addition in the TiO_2_/cellulose sample, which caused the subsequent photocatalytic degradation of MB. For all TiO_2_/chitin/cellulose composites with TiO_2_ (curve (f), (g), (h), and (I)), the concentrations of MB decreased more quickly than for the TiO_2_/cellulose sample. This demonstrates the function of chitin in enhancing the MB-removal capability of the samples. The MB concentration decreased to approximately 20%, 30%, and 40% of their original concentration after 2.5 h in the UV irradiation process for the samples with weight ratios of 5/2, 5/1, and 10/1 of cellulose to chitin, respectively. Therefore, by adding more chitin microparticles, i.e., from 0.5 g to 2 g, the 3D-printed samples demonstrated an improved photocatalytic capability. This can be explained by the fact that the structure and nature of TiO_2_ nanoparticles immobilized in the chitin/cellulose matrix could be better protected by an increased chitin/cellulose nano/microporous structure, a similar advantage to that observed with cellulose film matrix and TiO_2_ nanoparticles [43]. In addition, chitin has good adsorption capabilities. More chitin components added adsorption sites for MB molecules on the composites, which improved MB photocatalytic degradation efficiency. As for the chitin/cellulose composites without the TiO_2_ component, the curves in (c), (d), and (e) in Figure 7a show apparently slower decreasing rates than their counterpart samples containing TiO_2_ nanoparticles. The concentration of the samples without TiO_2_ could only be decreased to approximately 50% of their original concentrations, even after 10 h of the adsorption process. This indicates a good photocatalytic activity of TiO_2_ nanoparticles in the printed samples. Furthermore, more chitin components in the chitin/cellulose samples increased the MB-removal rate via adsorption. In summary, the dye removal capability of the 3D-printed cellulose sample was improved by the addition of TiO_2_ and further improved by adding more chitin. The formula of 5 g cellulose, 2 g chitin, and 0.3 g TiO_2_ (TiO_2_/chitin/cellulose (5/2)) produced the 3D-printed composite with the highest photocatalytic capability in the present study. It is worth noting that the pure TiO_2_ nanoparticles of 0.3 g exhibited the highest MB-removal capability, although it had a slower MB-removal rate over the first hours than the TiO_2_/chitin/cellulose samples. This is because TiO_2_ does not exhibit good MB adsorption [59]. Its MB-removal performance mainly depends on the photocatalytic degradation of MB.

To test the reusability of the 3D-printed TiO_2_/chitin/cellulose composite materials, five cycles of photocatalytic degradation of MB were performed after the TiO_2_/chitin/cellulose (5/2) sample had been restored by UV irradiation for 10 h and then freeze-dried before the following cycle. The curves showing the MB concentration changes for the five cycles are presented in Figure 7b. One sample with the same chitin/cellulose formula but without TiO_2_ was used in the same test as control. The sample without TiO_2_ was restored via 0.1 M HCl. Its curves are also presented in Figure 7b to demonstrate the reusability of the 3D-printed TiO_2_/chitin/cellulose composite materials for dye removal in wastewater. It was observed that the sample with TiO_2_ exhibited a similar performance in terms of completely removing MB in each cycle, while the capability of the sample without TiO_2_ decreased significantly in the following four cycles. This is because there are many MB molecules adsorbed on the cellulose/chitin surfaces via chemical interaction, and these are difficult to remove from the sample without degradation. The TiO_2_ nanoparticles can destroy the chemical interaction and degrade MB to restore the original structure of the chitin/cellulose surfaces. The mechanism for the restoration of TiO_2_/chitin/cellulose can be described using Equations (1)–(4) [35]:

(1) $$TiO_{2}+h\upsilon \;\to \;h_{VB}^{+}+e_{CB}^{-}$$

(2) $$e_{CB}^{-}+O_{2}\to \cdot O_{2}^{-}$$

(3) $$h_{VB}^{+}+H_{2}O\;\to \cdot OH+H^{+}$$

(4) $$O_{2}^{-}+\cdot OH+MB\to \;HCl+H_{2}SO_{4}+HNO_{3}+H_{2}O+CO_{2}$$

where $h_{VB}^{+}$ and $e_{CB}^{-}$ are the vacancy and the electron on the valence band and the conduction band of TiO_2_, respectively; ·OH and $\cdot O_{2}^{-}$ are hydroxyl radicals and superoxide radical anions, respectively. The electron-vacancy pairs generated in the reaction can result in ·OH and $\cdot O_{2}^{-}$ in water, which can finally oxidize and degrade MB molecules, as shown in Equation (4). Nevertheless, cellulose and chitin have high stability due to abundant inter/intra-hydrogen bonds among their molecules. The ·OH and $\cdot O_{2}^{-}$ generated in water by TiO_2_ are not sufficient to weaken the hydrogen bonds and dissociate cellulose and chitin. As such, the TiO_2_/chitin/cellulose composite photocatalyst is restored.

## 4. Conclusions

In this study, 3D printing using the NMMO method was applied to fabricate a chitin/cellulose matrix with a 3D ordered interconnected porous structure. It was used as a composite photocatalyst after being embedded with TiO_2_ nanoparticles. Anatase TiO_2_ nanoparticles were synthesized using a hydrothermal method. The characteristics of the 3D-printed TiO_2_/chitin/cellulose composite photocatalyst, such as the crystal phase, the specific surface area, and the photocatalytic activity, were significantly affected by the amount of chitin particles. The addition of chitin can weaken the intramolecular hydrogen bonding and reduce the crystallinity of the cellulose component in the composites. Furthermore, chitin slightly lowered the toughness of the pure cellulose, although the samples exhibited good strain characteristics before collapsing. The as-prepared TiO_2_/chitin/cellulose composites exhibited efficient photocatalytic activity in the degradation of MB dye solution under weak UV light irradiation, and excellent reusability and stability. As environmentally friendly products, 3D-printed TiO_2_/chitin/cellulose composite photocatalysts are portable, easily designed, and may be extended to various inorganic/cellulose functional materials for an array of potential applications.

## Figures and Tables

**Table 1:** Structural parameters of the 3D-printed TiO_2_/chitin/cellulose samples.

Sample	S_BET_ (m^2^/g)	Peaks of Pore Size (nm)	V_BJH_ (cm^3^/g)
TiO_2_	50	2.4	0.163
Cellulose	15	3.4	0.064
TiO_2_/Cellulose	1	2.3/3.4	0.004
TiO_2_/Chitin/Cellulose	3	7.7/30.8	0.018

## Data Availability

Not applicable.
